# Characterization of drug responses of mini patient-derived xenografts in mice for predicting cancer patient clinical therapeutic response

**DOI:** 10.1186/s40880-018-0329-5

**Published:** 2018-09-26

**Authors:** Feifei Zhang, Wenjie Wang, Yuan Long, Hui Liu, Jijun Cheng, Lin Guo, Rongyu Li, Chao Meng, Shan Yu, Qingchuan Zhao, Shun Lu, Lili Wang, Haitao Wang, Danyi Wen

**Affiliations:** 1 Shanghai LIDE Biotech Co., LTD, Shanghai, 201203 P. R. China; 2 Department of Surgery, Xijing Hospital, The Fourth Military Medical University, Xi’an, 710032 P. R. China; 3 Department of Oncology, Shanghai Chest Hospital Affiliated to Shanghai Jiao Tong University, Shanghai, 200030 P. R. China; 4 The Second Hospital of Tianjin Medical University, Tianjin Key Laboratory of Urology, Tianjin, 300211 P. R. China

**Keywords:** Personalized cancer therapy, Cancer precision medicine, Patient-derived xenograft (PDX), MiniPDX, Drug response, In vivo

## Abstract

**Background:**

Patient-derived organoids and xenografts (PDXs) have emerged as powerful models in functional diagnostics with high predictive power for anticancer drug response. However, limitations such as engraftment failure and time-consuming for establishing and expanding PDX models followed by testing drug efficacy, and inability to subject to systemic drug administration for ex vivo organoid culture hinder realistic and fast decision-making in selecting the right therapeutics in the clinic. The present study aimed to develop an advanced PDX model, namely MiniPDX, for rapidly testing drug efficacy to strengthen its value in personalized cancer treatment.

**Methods:**

We developed a rapid in vivo drug sensitivity assay, OncoVee^®^ MiniPDX, for screening clinically relevant regimens for cancer. In this model, patient-derived tumor cells were arrayed within hollow fiber capsules, implanted subcutaneously into mice and cultured for 7 days. The cellular activity morphology and pharmacokinetics were systematically evaluated. MiniPDX performance (sensitivity, specificity, positive and negative predictive values) was examined using PDX as the reference. Drug responses were examined by tumor cell growth inhibition rate and tumor growth inhibition rate in PDX models and MiniPDX assays respectively. The results from MiniPDX were also used to evaluate its predictive power for clinical outcomes.

**Results:**

Morphological and histopathological features of tumor cells within the MiniPDX capsules matched those both in PDX models and in original tumors. Drug responses in the PDX tumor graft assays correlated well with those in the corresponding MiniPDX assays using 26 PDX models generated from patients, including 14 gastric cancer, 10 lung cancer and 2 pancreatic cancer. The positive predictive value of MiniPDX was 92%, and the negative predictive value was 81% with a sensitivity of 80% and a specificity of 93%. Through expanding to clinical tumor samples, MiniPDX assay showed potential of wide clinical application.

**Conclusions:**

Fast in vivo MiniPDX assay based on capsule implantation was developed-to assess drug responses of both PDX tumor grafts and clinical cancer specimens. The high correlation between drug responses of paired MiniPDX and PDX tumor graft assay, as well as translational data suggest that MiniPDX assay is an advanced tool for personalized cancer treatment.

## Background

Genomic profiling has been widely applied in precision cancer medicine for molecularly stratified oncologic treatment. However, limitations in functional tests compromise the effectiveness of these tests in predicting responses to targeted therapies, hampering precision cancer medicine development. Integrating next-generation sequencing with functional assays, such as patient-derived tumor organoids and patient-derived tumor xenografts (PDXs), in testing drug responses has significantly improved the predictive power of these assays [1–3]. Recently, several studies have established the patient-derived tumor organoid model in various cancers, including gastrointestinal, bladder and breast cancer, and have shown a high predictive value of this model in assessing patient clinical response to targeted therapy or chemotherapy [4–6].

PDXs, by directly implanting patient tumor fragments into immunodeficient mice, have become critical in preclinical drug assessment as they capture the heterogeneity, and the molecular and histopathologic signatures of the parent primary tumors better than cell lines or genetically engineered mouse models. In addition, the drug response profiles of PDXs well correlate with patient clinical responses [3, 7–16]. PDXs have been reported in many different solid tumor types and have been proven useful in predicting patient chemotherapeutic response and providing guidance for informed clinical decision-making [9, 11, 16–22]. To date, approximately 300 cases of 13 tumor types have been evaluated and the overall concordance between patient clinical response and therapeutic response in PDXs ranges from 70 to 100%. Although PDXs possess notable advantages, limitations prevent their widespread utilization in personalized medicine. An unduly long period of time, usually 4–8 months, is required for tumor xenograft engraftment [8, 21, 23], and additional time is required to generate sufficient tissues for testing therapeutic regimens in mice. In addition, the engraftment rate in mouse models is generally lower than 50% in many cancer types, which is even lower for breast cancer, prostate cancer, and renal cell carcinoma [9, 15, 22]. Thus, many patients with rapid progressing disease could not benefit from PDX studies, and there is an urgent need for a fast and reliable alternative method to assess drug sensitivity.

The hollow fiber assay is used at the USA National Cancer Institute (NCI) as a preliminary screening tool for novel anticancer drugs [24]. This assay has certain advantages, simultaneous evaluation of compounds against various cell lines, relatively short term, low cost, and good correlation with conventional tumor graft assay [25, 26]. However, the limitation of using cell lines and lack of good correlation to clinical activity has historically hampered the usage of this approach.

By taking advantage of the hollow fiber implant technology, we sought to develop a fast and accurate in vivo drug response assay, which we named mini-patient-derived xenograft (MiniPDX) assay, to effectively and faithfully predict patient clinical response to targeted therapy and chemotherapy. We analyzed the histopathological and immunohistochemical features of tumor cells in MiniPDX capsules and compared the therapeutic responses of tumor xenograft in the MiniPDX model and the PDX model. The results altogether demonstrate that the MiniPDX assay offers a rapid and effective alternative approach to the PDX model in assessing cancer therapeutic responses that mimics patient clinical therapeutic responses.

## Materials and methods

### Tumor tissue acquisition

Fresh surgical tumor specimens were acquired from patients with pathologically proven gastric cancer, lung cancer or pancreatic cancer at participating hospitals. The list of participating hospitals will be provided upon written request. Cancer pathology was confirmed by an experienced pathologist (SY). The study protocol was approved by the Institutional Ethics Committee of Shanghai LIDE. Tumor tissue acquisition was approved by the ethics committees of each participating hospital and agreed to by each patient via written informed consent and was carried out according to state and institutional regulations on experimental use of human tissues.

### Animals

Six- to eight-week-old CB17-SCID or 5-week-old nu/nu mice (Charles River Co., Beijing, China) were housed at the AAALAC accredited animal facility at LIDE Biotech (Shanghai, China). CB17-SCID mice were used for PDX model recovery and nu/nu mice were used for drug efficacy tests. All study protocols were reviewed and approved by the Institutional Animal Care and Use Committee (IACUC) at LIDE Biotech, and conducted in accordance with established national and international regulations for laboratory animal protection.

### Establishing the PDX model

Fresh surgically removed gastric cancer (*n* = 14), lung cancer (*n* = 10) and pancreatic cancer tissues (*n *= 2) were used for establishing PDX models. Tumor cells were subcutaneously implanted into immune-deficient mice as previously described and stably propagated for three passages [8].

### Establishing the MiniPDX model

We developed an in vivo drug sensitivity MiniPDX assay by using a modified microencapsulation and hollow fiber culture system (OncoVee MiniPDX^®^, LIDE Biotech) according to the manufacturer’s instruction. Tumors ≥ 500 mm^3^ in size with a necrotic area < 30% were used. Briefly, tumor tissues were washed with Hank’s balanced salt solution (HBSS) to remove non-tumor tissues and necrotic tumor tissue in a biosafety cabinet. After the tumor tissues were morselized, they were digested with collagenase at 37 °C for 1–4 h. Cells were pelleted by centrifugation at 600*g* for 5 min followed by removal of blood cells and fibroblasts with magnetic beads. Cells were then washed with HBSS and filled into OncoVee^®^ capsules. Capsules were implanted subcutaneously via a small skin incision with 3 capsules per mouse (5-week-old nu/nu mouse).

### Histologic and immunofluorescence studies

Tumor tissues in the PDX assays and MiniPDX assays were fixed in buffered 10% formalin and routinely stained with hematoxylin and eosin (H&E) and examined by a certified pathologist.

For immunofluorescence studies, cellularized tumor cells (2 × 10^4^ cells, 200 L) were cytospun onto a slide, fixed with 4% paraformaldehyde for 20 min, permeabilized with 0.3% Triton X-100 in PBS for 30 min, and then blocked with 5% normal goat serum for 1 h at room temperature. The cells were then divided into three fractions and incubated with primary mouse monoclonal antibodies at 4 °C overnight against the following proteins: pan-cytokeratin, indicating carcinoma components [27, 28] (1:200, AE1/AE3, sc-81714, Santa Cruz Biotechnology, Santa Cruz, CA, US), *E*-cadherin, generally found in gastric adenocarcinomas [29] (1:50, HECD-1, ab1416, Abcam, Cambridge, UK), and MG7, a marker of gastric cancer [30] (1:300, NOTA-MG7) [30]. Subsequently, the cells were probed with secondary antibody donkey anti-mouse IgG H&L (Alexa Fluor^®^ 488) (1:200, ab150105, Abcam). Finally, the cells were mounted with DAPI-containing mounting medium (S36973, Thermo Fisher, MA, US). Images were captured with a fluorescence microscope (Leica, Germany) with Leica Application Suite V4 software and edited with Photoshop (Adobe, US).

### Pharmacokinetic assays

5-week-old nu/nu mice bearing MiniPDX capsules were administered orally with oxaliplatin (5 mg/kg) and approximately 200 μL blood was collected via a capillary in the retro-orbital plexus at different intervals post drug administration and directly mixed with 50 μL sodium citrate (3.8% solution). Blood samples were clarified by centrifugation and the supernatant was stored at − 80 °C. In addition, MiniPDX capsules were retrieved at indicated time intervals, morselized, and suspended in 500 μL PBS. After clarification by centrifugation at 1580*g* for 5 min, the supernatant was collected and stored at − 80 °C. The concentrations of oxaliplatin in the plasma and the MiniPDX capsules were analyzed by LC–MS/MS and pharmacokinetic parameters were calculated using the WinNonlin^®^ 6.4 program.

### MiniPDX drug sensitivity assays

Mice bearing MiniPDX capsules were treated with appropriate drugs or their combinations as detailed in Tables 1 and 2 for 7 days. Thereafter, the implanted capsules were removed and tumor cell proliferation was evaluated using the CellTiter Glo Luminescent Cell Viability Assay kit (G7571, Promega, Madison, WI, US) as instructed by the manufacturer. Luminescence was measured in terms of relative luminance unit (RFU) using a spectrophotometer (SpectraMax M3, Molecular Devices, Sunnyvale, CA, US). Tumor cell growth inhibition (TCGI) (%) was calculated using the formula:

$${\text{TCGI }}\left( \% \right) \, = \, \left( \begin{aligned} 1- \left[ {{\text{Mean RLU of the treatment group on day 7}} - {\text{ Mean RLU on day }}0} \right) \hfill \\ /\left( {{\text{Mean RLU of the vehicle group on day 7}} - {\text{Mean RLU on day }}0} \right] \hfill \\ \end{aligned} \right) \, \times 100\%$$

Each experiment was done in sextuplicate and mean values were reported. A positive drug response was considered present if TCGI was ≥ 45% (*P* < 0.05), and a negative drug response was considered if TCGI was < 45% (*P* < 0.05).

**Table 1:** Drug preparations and treatment details

Drug	Supplier	Preparation^a^	PDX assay^b^	MiniPDX assay^b^
S-1	Hengrui	0.5% HPMC + 0.2% Tween 80	10 mg/kg, *po*, qd*5/w	10 mg/kg, *po*, qd*5
Docetaxel	DEMO	5% Tween 80 + 5% Ethanol + 90% Saline	20 mg/kg, *ip*, q4d	20 mg/kg, *ip*, q4d*2
Gemzar	Eli Lilly	Saline	60 mg/kg, *ip*, q4d	60 mg/kg, *ip*, q4d*2
Oxaliplatin	Hengrui	5% Glucose	5 mg/kg, *ip*, biw	5 mg/kg, *ip*, q4d*2
Irinotecan	DEMO	5% DMSO + 95% Saline	40 mg/kg, *ip*, q4d	50 mg/kg, *ip*, q4d*2
Cisplatin	Hansoh	Saline	5 mg/kg, *ip*, qw	5 mg/kg, *ip*, q4d*2
Epirubicin	Pfizer	Saline	5 mg/kg, *ip*, qw	5 mg/kg, *ip*, q4d*2
Capecitabine	Adamas	0.5% HPMC + 0.2% Tween 80	400 mg/kg, *po*, qd*14	400 mg/kg, *po*, qd*7
5-FU	Xudong-Haipu	Saline	25 mg/kg, *ip*, qd*5/w	25 mg/kg, *ip*, qd*5
Erlotinib	Topscience	0.5% HPMC + 0.2% Tween 80	50 mg/kg, po, qd	50 mg/kg, po, qd*7
Crizotinib	Aladdin	0.5% HPMC + 0.2% Tween 80	50 mg/kg, po, qd	50 mg/kg, po, qd*7
AZD9291	Topscience	0.5% HPMC + 0.2% Tween 80	5 mg/kg, po, qd	5 mg/kg, po, qd*7

*po* oral, *ip* intraperitoneal, *qd* once a day, *biw* twice a week, *qw* once a week, *q4d* once every 4 days

^a^ Recipe of formulation

^b^ Dose, dosing route, dosing frequency followed by, where indicated, dosing times and/or treatment duration

**Table 2:** Treatment details of combination regimens

Regimen	Drug 1	Drug 2	Drug 3
2	S-1 (6.9 mg/kg, *po*, qd*14)	Oxaliplatin (5 mg/kg, *ip*, qw)	NA
3	Capecitabine (400 mg/kg, *po*, qd*14)	Oxaliplatin (5 mg/kg, *ip*, qw)	NA
4	Capecitabine (400 mg/kg, *po*, qd*14)	Oxaliplatin (5 mg/kg, *ip*, qw)	Epirubicin (5 mg/kg, *ip*, qw)
5	Cisplatin (5 mg/kg, *ip*, qw)	5-FU (15 mg/kg, *ip*, qd*5)	Docetaxel (20 mg/kg, *ip*, qw)
7	Gemzar (60 mg/kg, *ip*, q4d)	Cisplatin (5 mg/kg, *ip*, qw)	NA
12	Oxaliplatin (5 mg/kg, *ip*, qw)	Irinotecan (40 mg/kg, *ip*, q4d)	NA

Drug combinations used to test efficacy in PDX models, including detailed treatment conditions in brackets (); Combination regimens have the same numbering as Table 3

*NA* not available, *po* per os, *ip* intraperitoneal, *qd* once a day, *biw* twice a week, *qw* once a week, *q4d* once every 4 days

### Evaluation of therapeutic responses

The therapeutic response of primary tumors in PDX models to 12 clinically relevant regimens, including 9 chemotherapeutic drugs and 3 targeted drugs was examined (Table 3). Tumor volume was measured by a caliper twice a week and calculated as (length × width^2^)/2, and tumors were harvested when they reached 500–700 mm^3^ and were morselized and snap-frozen in liquid nitrogen. Morselized tumors were inoculated in the right flank of nu/nu mice and when they reached 100–300 mm^3^, mice were randomized to receive vehicle or indicated regimens for 3 weeks as detailed in Tables 1 and 2. Antitumor efficacy was represented by tumor growth inhibition (TGI) (%) and calculated using the formula:

$${\text{TGI }}\left( \% \right) \, = \, \left[ { 1- \left( {{\text{V}}_{\text{ti}} - {\text{V}}_{{{\text{t}}0}} } \right)/ \, \left( {{\text{V}}_{\text{ci}} - {\text{V}}_{{{\text{c}}0}} } \right)} \right] \, \times 100\%$$

where V _t0_ and V_ti_; and V_c0_ and V_ci_ were the tumor volume at the first day of drug or vehicle treatment and the final tumor volume in the treatment group and the control group, respectively. The cutoff of TGI ≥ 45% (*P* < 0.05) was used to define positive response, and TGI < 45% (*P* < 0.05) was used to define negative response.

**Table 3:** Drug efficacy in PDX models and OncoVee^®^ MiniPDX capsules in mice

Model	Location	Pathology	Chemotherapeutic or targeted drug (Regimen)	TGI (%)	Response in PDX	TCGI(%)	Response in MiniPDX
GAYW5	Stomach	Poor/moderately differentiated AC, 80%	S-1 (1)	95 ± 6	+	94 ± 15	+
GAYW7	Stomach	Poorly differentiated AC, 90%	S-1 (1)	86 ± 10	+	92 ± 3	+
GAYL1	Stomach	Mucinous AC, 80%	S-1 (1)	37 ± 10	–	13 ± 29	–
GAYB7	Stomach	Poorly differentiated tubular AC, 70%	S-1 (1)	44 ± 16	–	14 ± 20	–
GAYP53	Stomach	Poor-moderately differentiated AC, 90%	S-1 (1)	35 ± 10	–	17 ± 13	–
GASIL2	Stomach	Moderately differentiated AC, 40%	S-1 + Oxaliplatin (2)	29 ± 20	–	40 ± 17	–
GABSI3	Stomach	Poorly differentiated AC, 80%	S-1 + Oxaliplatin (2)	37 ± 12	–	75 ± 4	+
GAYP93	Stomach	Moderately differentiated AC, 90%	S-1 + Oxaliplatin(2)	27 ± 20	–	29 ± 15	–
GAYP97	Stomach	Moderately differentiated AC, 90%	S-1 + Oxaliplatin (2)	7 ± 29	–	9 ± 10	–
GAJ07	Stomach	Poor-moderately differentiated AC, 50%	Capecitabine + Oxaliplatin (3)	80 ± 3	+	35 ± 13	–
GASI80	Stomach	Poorly differentiated AC, 80%	Capecitabine + Oxaliplatin (3)	112 ± 3	+	50 ± 11	+
GASI05	Stomach	Moderately differentiated AC, 40%	Epirubicin + Capecitabine + Oxaliplatin (4)	97 ± 16	+	45 ± 23	+
GASAB3	Stomach	Poorly differentiated AC, 90%	Cisplatin + 5-FU + Docetaxel (5)	43 ± 17	–	17 ± 14	–
GAYP16	Stomach	Poorly differentiated AC, 90%	Cisplatin + 5-FU + Docetaxel (5)	32 ± 16	–	−13 ± 39	–
GAYP53	Stomach	Poor-moderately differentiated AC, 90%	Cisplatin + 5-FU + Docetaxel (5)	125 ± 3	+	50 ± 7	+
LULI02	Lung	Poorly differentiated SCC, 98%	Docetaxel (6)	97 ± 12	+	51 ± 7	+
LULI03	Lung	Poorly differentiated AC, 98%	Docetaxel (6)	12 ± 23	–	15 ± 10	–
LULI20	Lung	Poorly differentiated AC, 98%	Docetaxel (6)	91 ± 14	+	86 ± 3	+
LULI21	Lung	Poorly differentiated SCC, 78%	Docetaxel (6)	42 ± 16	–	−61 ± 29	–
LULI27	Lung	Moderate-highly differentiated SCC, 80%	Docetaxel (6)	115 ± 3	+	32 ± 11	–
LULI55	Lung	Large cell carcinoma, 95%	Docetaxel (6)	109 ± 1	+	14 ± 4	–
CTYW012	Lung	Poor-moderately differentiated AC, 90%	Gemzar + Cisplatin (7)	116 ± 0	+	84 ± 7	+
LULI49	Lung	Poorly differentiated AC, 90%	Erlotinib (8)	40 ± 15	–	−16 ± 26	–
CTC15063	Lung	Poor-moderately differentiated AC, 95%	Erlotinib (8)	37 ± 19	–	21 ± 17	–
CTC15063	Lung	Poor-moderately differentiated AC, 95%	AZD9291 (9)	167 ± 5	+	61 ± 3	+
CTC16075	Lung	Poorly differentiated carcinoma, 90%	Crizotinib (10)	103 ± 2	+	102 ± 4	+
PAYY8	Pancreas	Poorly differentiated ductal AC, 90%	Gemzar (11)	27 ± 27	–	−14 ± 10	–
PAYY5	Pancreas	Poor-moderately differentiated ductal AC, 80%	Gemzar (11)	75 ± 9	+	56 ± 8	+
PAYY5	Pancreas	Poor-moderately differentiated ductal AC, 80%	Oxaliplatin + Irinotecan (12)	112 ± 2	+	54 ± 6	+

Model: Indicates a specific patient and patient-derived xenograft model

Pathology: Judged by licensed pathologist (SY); %, percentage of the diseased cells judged by pathology; *AC* adenocarcinoma, *SCC* squamous cell carcinoma

Regimen: Drug combinations used to test efficacy in specific PDX model and MiniPDX

Chemotherapeutic or targeted drug: Single or combination of drugs used in PDX assay and in MiniPDX

TGI: Tumor growth inhibition or TCGI: tumor cell growth inhibition. N = 6, results are mean ± SEM. Mean TGI or TCGI ≥ 45% is defined as positive therapeutic response (+)

### Statistical analysis

Statistical data and graphics were analyzed using GraphPad Prism 6. Statistical significances were assessed by Student’s *t* test with *P* < 0.05 considered significant. The positive predictive value (PPV) was calculated using the formula:

$${\text{PPV }} = {\text{ No}}.{\text{ of true positives}}/{\text{No}}.{\text{ of true positives }} + {\text{ No}}.{\text{ of false positives}} \times 100\%$$

and the negative predictive value (NPV) was calculated using the formula:

$${\text{NPV }} = {\text{ No}}.{\text{ of true negatives}}/{\text{No}}.{\text{ of true negatives }} + {\text{ No}}.{\text{ of false negatives}} \times 100\%.$$

## Results

### The MiniPDX model could be used to assess therapeutic response of primary tumor cells

**Fig. 1:**
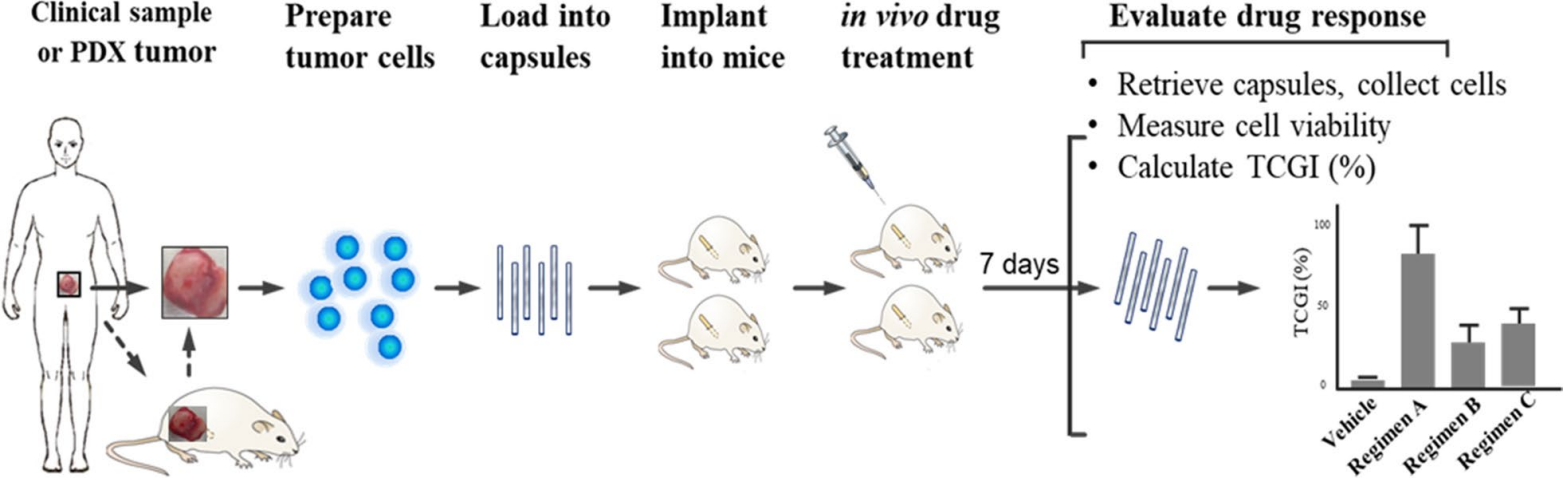
Development of OncoVee^®^ MiniPDX Assay for rapid systemic detection of drug sensitivity in vivo. Also see details in Methods

**Fig. 2:**
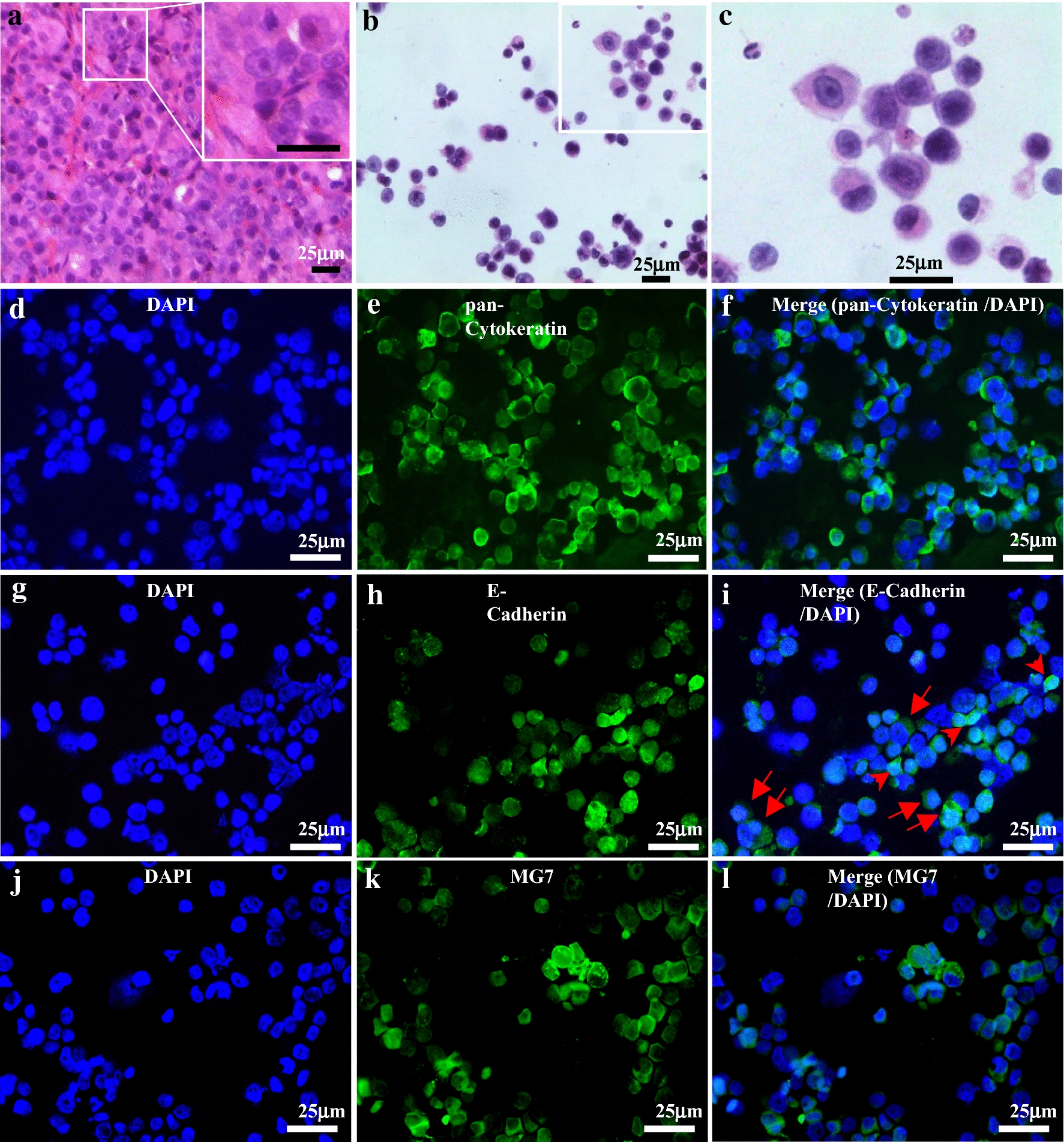
Morphologic and immunohistochemical features of cells retrieved from the implanted capsules in MiniPDX-bearing mice. **a** Tissue section of a PDX xenograft tumor (GASI80) showing typical feature of poorly differentiated adenocarcinoma; inlet: High magnification view revealing tightly arranged poorly differentiated cells. **b**, **c** Cytospin of cells retrieved from the capsules implanted in MiniPDX-bearing mice, low- and high-power view, respectively (H&E stain), showing that the majority of the cells are associated with high nucleus to cytoplasm ratio, hyperchromatic nuclei, and scant cytoplasm. Immunofluorescent staining of pan-cytokeratin (**e**), *E*-cadherin (**h**) and MG7 (**i**); 4′, 6-diamidino-2-phenylindole staining for individual panels (**d**, **g**, **j**). Merged images (**f**, **i**, **l**) show that the cells cultivated within the OncoVee^®^ capsules expressed all of the three characteristic primary gastric cancer-related markers. Scale bars, 25 μm. The tumor cells cultivated in the MiniPDX capsules, which were derived from PDX tumor of gastric adenocarcinomas (PDX model GASI80, **a** H&E stain of tissue section), strongly expressed pan-cytokeratin (**e**, **f**) *E*-cadherin (**h**, **i**), and MG7 (**k**, **l**)

We developed a rapid in vivo drug sensitivity assay, the MiniPDX assay, for assessing therapeutic response of primary tumor cells. The integral process of MiniPDX assay included from sample preparation to drug response evaluation (Fig. 1). Our histologic and immunohistochemical study revealed that cells from the MiniPDX assay were morphologically and immunohistochemically similar to their original primary cancer cells (Fig. 2a, b–i), suggesting that tumor cells within the MiniPDX capsules closely mimic their parental primary tumor cells.

**Fig. 3:**
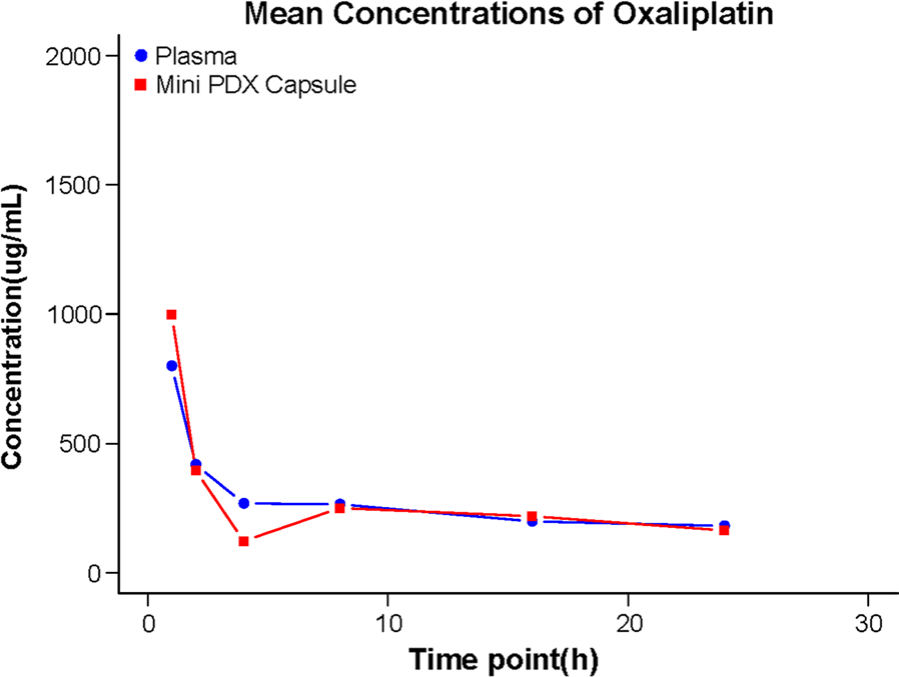
Pharmacokinetic results of oxaliplatin in MiniPDX capsule and in plasma. The mean concentration of oxaliplatin in the MiniPDX capsule and in plasma was estimated by LC–MS/MS

We further evaluated the dynamic changes of drug concentration of orally administered oxaliplatin in the MiniPDX capsules. We found that drug in MiniPDX capsules turned out essentially the same as that in plasma (Fig. 3), indicating that the capsules do not limit in vivo distribution of oxaliplatin and the MiniPDX model could be used to assess systemically administered drugs.

### The MiniPDX model and the PDX model exhibit largely consistent therapeutic responses

To further evaluate the consistency of therapeutic responses between MiniPDX assay and PDX model, we compared the therapeutic responses of 26 randomly selected primary tumors in the PDX model and the MiniPDX model (Table 3). If the therapeutic responses in PDX model and corresponding MiniPDX assay were both positive or negative, they would be defined as consistent therapeutic responses. Twelve (85.7%, 12/14) gastric cancer tissues showed consistent therapeutic responses to 15 drugs in the PDX model and the MiniPDX model. Five gastric cancer tissues had a TGI and TCGI ≥ 45% and eight gastric cancer tissues had a TGI and TCGI < 45% in both the PDX model and the MiniPDX model, including one lung cancer tissue (GAYP53) with a TGI and TCGI ≥ 45% to cisplatin plus 5-FU and docetaxel, and a TGI and TCGI < 45% to S-1 in both the PDX model and the MiniPDX model. Similarly, 8 (80%, 8/10) lung cancer tissues showed consistent therapeutic responses in the PDX model and the MiniPDX model. Five lung cancer tissues had a TGI and TCGI ≥ 45% and 4 lung cancer tissues had a TGI and TCGI < 45% in both the PDX model and the MiniPDX model, including one lung cancer tissue (CTC15063) with a TGI and TCGI ≥ 45% to AZD9291 and a TGI and TCGI < 45% to erlotinib in the PDX model and the MiniPDX model. Two (100%, 2/2) pancreatic adenocarcinoma tissues were fully consistent in therapeutic responses in the MiniPDX model and the PDX model.

### The MiniPDX model could predict clinical response of cancer patients

**Fig. 4:**
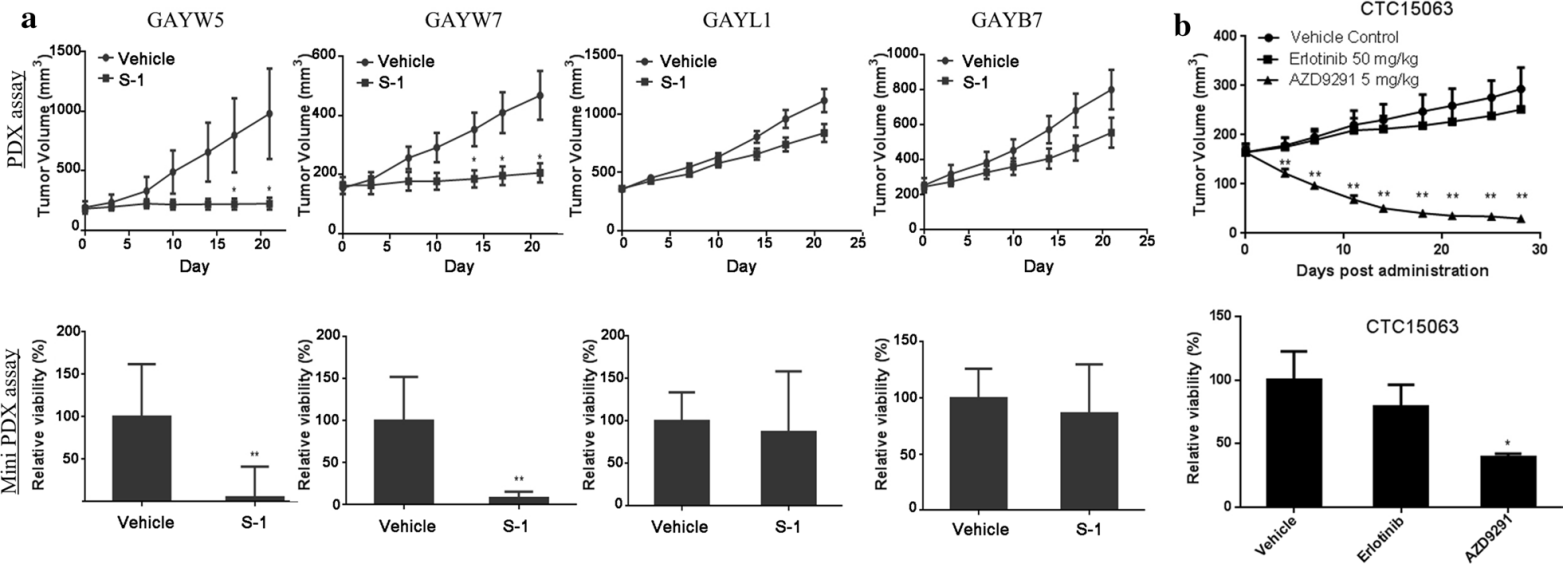
Comparing the efficacy results in MiniPDX assays and results in PDX assays. **a** Representative results of pairwise efficacy tests in 4 PDX xenograft models against S-1 regimens in the same row (See Table 3 for detailed information). Note: Similar results were observed in MiniPDX assays (lower panel, tumor cell viability) as those in PDX assay (upper panel, tumor growth curve). After the treatment, GAYW5 and GAYW7 showed a marked decrease in tumor volume or cell viability but GAYL1 and GAYB7 did not. **b** Pairwise efficacy test in Model CTC15063 against erlotinib and AZD9291 in the same row. The efficacy results of MiniPDX (lower panel, tumor cell viability) are consistent with those in PDX models (upper panel, tumor growth curve). (*n* = 6, values showing are mean ± SEM; *P < 0.05; **P < 0.01 in comparison to the vehicle group, paired Student’s *t* test)

We further examined the therapeutic response of 4 gastric cancer tissues (GAYW5, GAYW7, GAYL1 and GAYB7) with known clinical responses to S-1. PDX assays showed that S-1 caused a significantly greater reduction in the tumor volume of GAYW5 and GAYW7 than vehicle control while no difference in tumor volume was seen in GAYL1 and GAYB7 between S-1 and control (*P* < 0.05) (Fig. 4a). The miniPDX assays further showed that S-1 significantly reduced the viabilities of GAYW5 and GAYW7 (*P* < 0.001) while no difference in the viabilities of GAYL1 and GAYB7 was seen (Fig. 4a). The results of the miniPDX assays are consistent with the findings of the PDX assays and the clinical response of the patients. The genomic sequencing data, therapeutic response from the PDX model and clinical response of lung cancer CTC15063 were previously published [31]. Consistently, CTC15063 showed greater sensitivity to AZD9291 than erlotinib in terms of tumor volume reduction in the PDX assays but significantly lower viabilities in response to erlotinib in the PDX assays (Fig. 4b).

In addition, overall 29 pairwise efficacy tests were conducted on 26 PDX xenografts against 12 therapeutic regimens. Compared against the PDX assay, the MiniPDX assay had a PPV of 92%, a NPV of 81%, and a sensitivity of 80% and a specificity of 93%, suggesting that the MiniPDX assay is of high predictive power.

### Application of MiniPDX assay in clinical settings

**Fig. 5:**
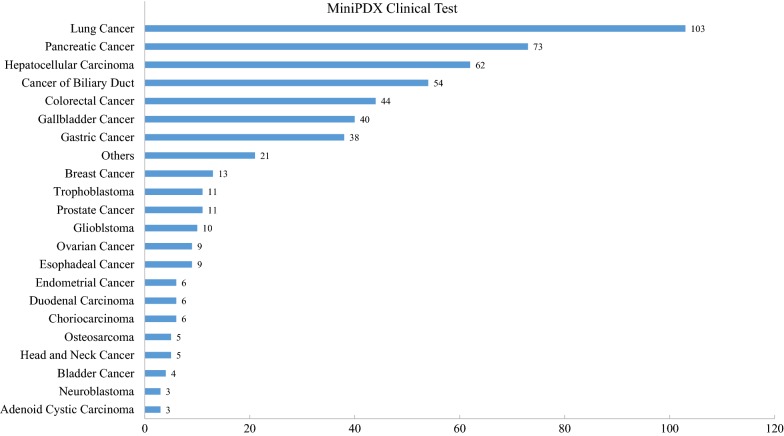
Establishment of MiniPDX models in clinical various cancer. Establishment of MiniPDX models and treatment regimen selection using clinical specimens. X-axis represents the number of MiniPDX models in each malignancy type; Y-axis represents the tumor type

We also continuously looked into MiniPDX assay data using patients’ tumor specimens. To date, 536 clinical samples comprising up to 40 malignancy types were obtained (Fig. 5). Four hundred twenty samples (79%) passed the quality control criterion and underwent MiniPDX tests. Quite interestingly, the MiniPDX assay yielded a 100% success rate achieved with these qualified samples.

### Case report

**Fig. 6:**
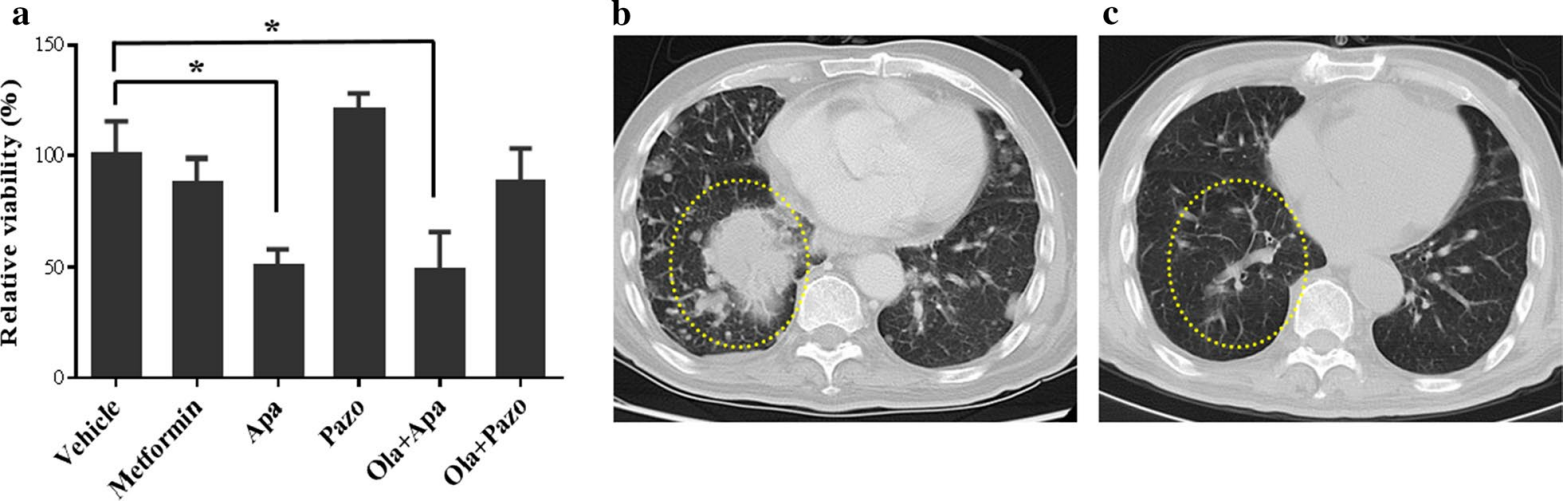
Application of MiniPDX assay in clinical setting. Representative clinical case study of MiniPDX assay in patient MDX245, with bilateral multiple pulmonary metastases from low grade endometrial stromal sarcoma. **a** Response of MDX245 patient’s MiniPDX model to single agent apatinib and combined apatinib with olapanib. (*n* = 6; *, *P *< 0.05 in comparison to the vehicle group); **b**, **c** Chest CT scans of patient MDX245, before and 4 months after treatment. Note a significant eradiation in bilateral multiple masses including a massive one (dotted oval). *Ola* olapanib, *Apa* apatinib, *Pazo* pazopanib

Patient MDX245, a 48-year-old female, presented with bilateral multiple pulmonary metastases from low grade endometrial stromal sarcoma. She was initially treated with laparoscopic surgery and a regimen including lobaplatin, doxorubicin and ifosfamide. After only 2 cycles of chemotherapy, her disease progressed in the lungs and severe myelosuppression developed. Clinical investigation indicated that the patient could be a candidate for apatinib therapy. The MiniPDX tests with 4 different targeted drugs in 5 regimens showed that the lung metastasis responded to single agent apatinib and apatinib in combination with olapanib, but not to metformin, pazopanib or pazopanib combined with olapanib (Fig. 6a). Indeed, 4 months post treatment, the patient achieved partial regression in her lung metastases that lasted for 8 months (Fig. 6b, c). The patient was currently being followed up.

## Discussion

Experimental in vivo models that closely mimic the biology of cancer in patients are urgently needed to reliably predict optimal sensitivities to available regimens in personalized chemotherapy [9, 11, 16–22]. Such models can yield drug test results within a short time frame to guide prompt cancer therapy [32–35]. The present study demonstrated that the MiniPDX sensitivity assay using fresh tumor samples is a rapid and effective alternative to the PDX model in capturing therapeutic responses of primary tumor tissues that mimic patient clinical therapeutic response and is of high predictive power with a sensitivity of 80% and a specificity of 93%.

The entire process of MiniPDX assay to test patient tumor response to chemotherapeutic or targeted drugs in immunodeficient mice can be completed within 7 days. This is in contrast to duration of 4–8 months required for the PDX assay [8, 21, 23]. Recently, many patient-derived culture models such as patient-derived tumor organoid [4–6] and xenograft models have been utilized as rapid functional testing tools to predict therapeutic response. However, these ex vivo methods cannot mimic in vivo therapeutic response to systemically administered drugs in patients [36]. In addition, certain drugs can only be tested in vivo, not in vitro, as they undergo physiological metabolism before they become active. Thus, in vitro culturing assays such as patient derived tumor organoid cannot meet the need. In contrast, MiniPDX assays take the advantage of in vivo growth condition, which involves 3-dimentional growth (such as that in organoids), tumor microenvironment and tumor heterogeneity. Meanwhile, Mini-PDX-bearing mice received systemic drug administration as under clinical conditions. Thus, the MiniPDX assay is well positioned for wide clinical application in cancer precision medicine.

Streamlined conditions in MiniPDX assays allowed in vivo survival and growth of tumor cells, especially primary tumor cells of various cancer types, thus yielding a high success rate. According to our extensive MiniPDX studies, as long as quality control was met, a 100% success rate could be achieved using either PDX tumor grafts or surgically resected tumors, biopsy and thoracentesis specimens (Table 3 and Fig. 5a). The MiniPDX assay does not require prior PDX model establishment, which, a pre-requisite in in vivo PDX assay, often takes several months, and the model establishment rate is generally much lower than 50% [9, 15, 22].

Tumor cells in the MiniPDX assays closely resemble parental primary tumors or original PDX tumors with regard to cancer type-specific morphologic and immunohistochemical features, as well as overall tumor heterogeneity (Fig. 2). During MiniPDX modeling tumor cells do not undergo any pressure selection like PDX tumor does during tumor engrafting in a host animal.

In the MiniPDX model, tumor cells were suspended in culture media and filled in implant capsules, which are made of hollow fiber membrane with a 500 kD pore size allowing molecules less than 500 kD to move in and out freely while keeping cells within the capsule. The surface of the implant membranes has been shown to be biocompatible in various animal models for periods exceeding 14 days [25, 26]. Recent studies using visualizing tools have demonstrated that tumor cells inside the fibers behave properly, including secreting cellular factors, communicating with the host mice and generating angiogenesis around cell-filled fibers. Furthermore, the fiber system delivers media to the cells in a manner akin to the delivery of blood through the capillary networks in vivo [37–39]. In addition, earlier studies of cell lines grown within the capsule, followed by implantation into a host animal, showed that human tumor cells are not subjected to host immunological attack [24]. In our streamlined conditions of MiniPDX assays, we observed that viability of untreated cells generally increased to 3 to 5 times after 7 days. Thus, MiniPDX assay holds high potential to evaluate various anticancer agents including antibody drugs. Collectively, tumor cells in MiniPDX are highly similar to original cancer cells with respect to phenotypic as well as molecular properties [27–30]. Clearly, the MiniPDX models used in this study are closely related to original malignancies, and assays based on such models for predicting drug responses would be highly relevant to the clinical situation.

For personalized chemotherapy, testing chemosensitivity for a few different chemotherapeutic regimens is desired for individual cancer patients. We have empirically determined that testing three drugs in Mini-PDX would require > 500 mm^3^ tumor size and ≥ 70% tumor cell viability. The vast majority of cancer patients in which adjuvant chemotherapy is indicated according to the tumor-node-metastasis stage, have primary tumors of > 2 cm in diameter and thus, providing sufficient tumor tissue for standard pathologic examination as well as for testing a variety of regimens in MiniPDX assays.

In summary, we developed, streamlined and validated a rapid systemic in vivo MiniPDX assay to predict clinical outcome. We systematically evaluated and compared the response rates of PDX assays and MiniPDX assays (Table 3) pair-wise in 26 PDX models of 3 types of cancers to 12 clinical relevant regimens, and we found a high correlation between drug responses of the two assays (Table 4). Although the sample tests were limited, our results indicated that the differential correlation response of the two assays could be present in different cancer types. We also confirmed this rapid testing method is feasible for various clinical samples to guide clinical treatment. Through a representative case of metastatic cancer patient, we demonstrated that clinical benefit was achieved using MiniPDX sensitivity results to guide clinical treatment.

**Table 4:** Correlation response of MiniPDX *versus* PDX assays in PDX models

		Response in PDX		
		R	NR	Total
Response in Mini-PDX				
R		12	1	13
NR		3	13	16
Total		15	14	29
Positive predictive value				92%
Negative predictive value				81%
Sensitivity				80%
Specificity				93%

R: responder; NR: non-responder

Considering the current limited sample size and probable differential correlation response in different cancer types, several PI-initiated clinical trials for real world evidence (RWE) studies with FDA part 11 in compliance are registered and in progress for further evaluating the correlation between MiniPDX and clinical responses in a wide range of cancer types. The clinical effectiveness of proposed treatments, including determination of patient objective response rates, progression-free survival, and adverse effects, will be essential for expanding clinical usage of MiniPDX assays in personalized cancer precision treatment.

## Conclusions

We developed a fast, systemic in vivo drug sensitivity assay, namely MiniPDX, in which patient primary tumor cells are capsulated and implanted in mouse to reliably and precisely test tumor responses to different antitumor drugs. MiniPDX method, as a complementary, if not an alternative, approach to PDX assay, is suitable for fast drug response assessment of primary cancer cells in order to select effective or to spare non-responding therapeutic regimens. MiniPDX holds promise to aid personalized therapy of cancer patients.

## Abbreviations

PDX: patient-derived xenograft
MiniPDX: mini-patient-derived xenograft
TGI: tumor growth inhibition
TCGI: tumor cell growth inhibition
RLU: relative luminance unit
NCI: National Cancer Institute
RWE: real world evidence
IACUC: the Institutional Animal Care and Use Committees
H&E: hematoxylin and eosin
MEHFC: microencapsulation and hollow fiber culture
AC: adenocarcinoma
SCC: squamous cell carcinoma
LCC: large cell carcinoma
Ola: olapanib
Apa: apatinib
Pazo: pazopanib
*po*: oral
*ip*: intraperitoneal
qd: once a day
biw: twice a week
qw: once a week
q4d: once every 4 days
